# Rare Location of Osteoid Osteoma at the Olecranon: A Case Report

**DOI:** 10.7759/cureus.77513

**Published:** 2025-01-15

**Authors:** Dimitrios Kotzamitelos, Maria Tsatlidou, Alexandros P Tzaveas, Michael Iosifidis

**Affiliations:** 1 3rd Orthopaedic Department, Interbalkan Medical Center, Thessaloniki, GRC

**Keywords:** elbow pain, excision of tumour, intraarticular lesion, nidus, olecranon tip, osteoid osteoma

## Abstract

A rare case of an elbow osteoid osteoma located at the tip of the olecranon in a 20-year-old male martial arts athlete is presented. To the best of our knowledge, it is the first time this location of an osteoid osteoma has been reported in the literature. Diagnosis may be challenging; however, the excision of the lesion may lead to complete recovery.

## Introduction

Osteoid osteoma is a small benign osteoblastic tumour with a characteristic core (nidus) and a reactive sclerotic surrounding zone in radiographs [1]. It is more commonly reported in young males within the first three decades of life [2]. The tumour typically presents with an insidious onset of pain that is worse at night and relieves by non-steroidal anti-inflammatory medications (NSAIDs). It is found frequently in the diaphysis of long bones such as the femur and tibia [3]. Intraarticular or very close to joint locations are considered to be rare entities [4,5].

Intraarticular elbow osteoid osteomas have been reported mostly in the olecranon fossa of the distal humerus [6-9]. Generally, they represent a diagnostic challenge as they can mimic other common pathologies such as osteoarthritis or tendinitis [10,11], and this can cause a delay in the diagnosis.

A case of a symptomatic osteoid osteoma at the tip of the olecranon process is described, and to the best of our knowledge, this location is very rarely reported in the literature.

## Case presentation

**Figure 1:**
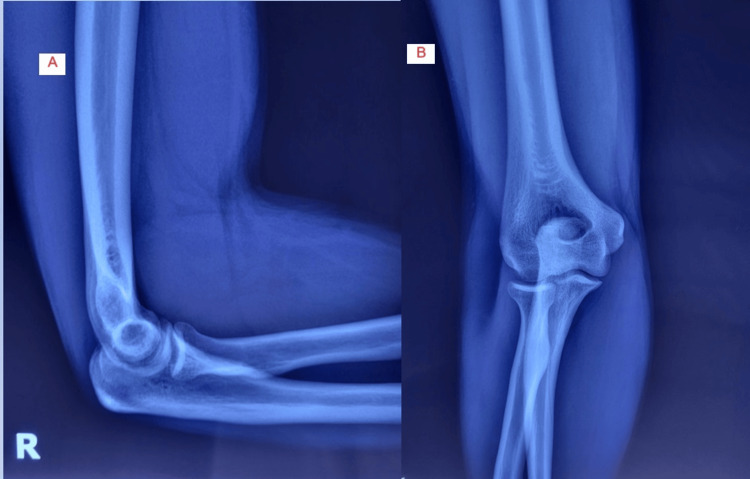
Initial radiological Imaging. Standard anteroposterior (A) and lateral (B) views with no pathological findings.

A 20-year-old male (right-handed) student with a BMI of 24.8 and a keen athlete of taekwondo presented with pain in the right elbow. Apart from his intense athletic activities, there was no distinct injury in his clinical history. From his general health condition history, Crohn's disease under treatment was referred with negative previous surgical history. The patient complained of posterior elbow pain during activities as well as occasional discomfort during rest. No nocturnal pain was referred. Clinical examination revealed a normal range of motion and mild tenderness over the attachment of the triceps tendon. Initial treatment as tendinopathy with rest and analgesia had a moderate effect on the pain. It is worth mentioning that due to his inflammatory bowel disease, NSAIDs were not allowed. Six weeks later, an anteroposterior and lateral X-ray of the elbow revealed no pathologic findings (Figure 1).

**Figure 2:**
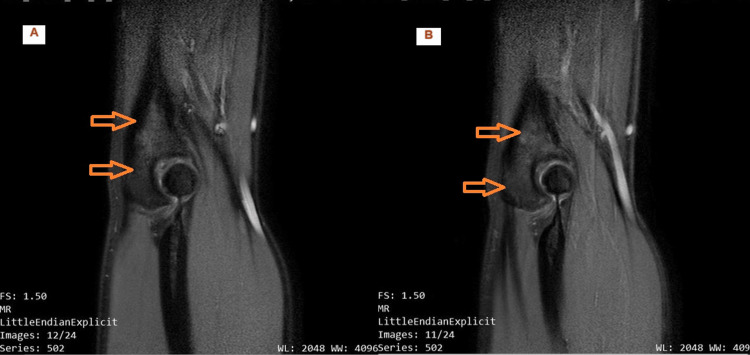
Two sequential sagittal images on first MRI. (A and B) Diffuse bone marrow oedema of distal humerus two months after the onset of symptoms.

**Figure 3:**
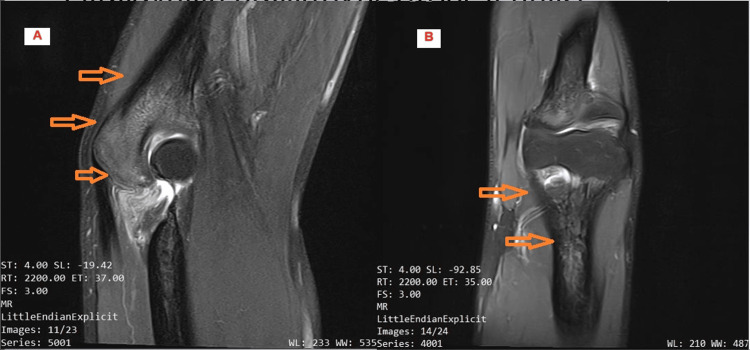
Second MRI. Sagittal (A) and coronal (B) views on persistent bone marrow oedema (arrows) three months after the first MRI and after conservative treatment.

A second course of conservative treatment was followed with rest, ice therapy, painkillers, and physiotherapy. After two months, the symptoms were milder but still existed; the patient had intermittent sports activity, and an MRI scan was performed (Figure 2). Diffuse bone edema was observed in the olecranon with no evidence of a fracture. There was also mild subcutaneous oedema in the ulnar aspect of the olecranon and the rest of the elbow structures were observed normal. A possible diagnosis of enthesopathy was made as there was no direct injury to the olecranon. Injection treatment was proposed with corticosteroids at first. After its failure to control the symptoms, two weeks later, a platelet-rich plasma (PRP) injection was performed (three courses at two-week intervals), alongside affected arm suspension and physiotherapy courses in between the injections. A second MRI five months after the onset revealed no radiological improvement, with the bone oedema of the olecranon being the main finding observed (Figure 3).

**Figure 4:**
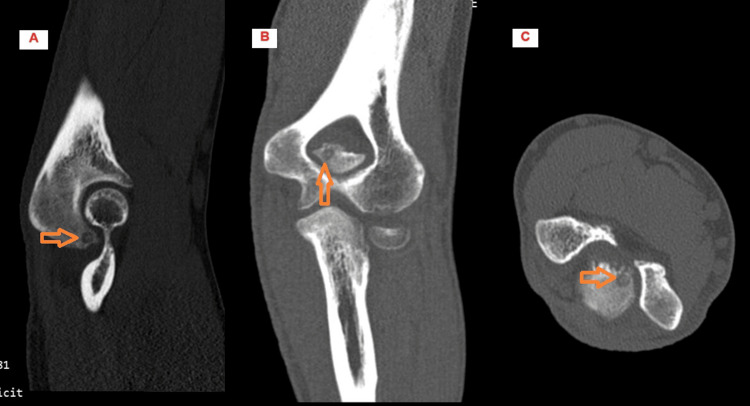
CT Scans of Elbow Sagittal (A), coronal (B), and axial (C) views of osteoid nidus located at olecranon tip (arrows) shown in elbow CT images ten months after onset of symptoms.

In the absence of a fracture, the decision was made to attempt treatment of the bone oedema with hyperbaric O_2_ therapy sessions (20 sessions). Nearly ten months after the onset, the patient was still symptomatic. Finally, a third MRI scan demonstrated for the first time a nidus of approximately 5 mm diameter at the tip of the olecranon surrounded by bone oedema. The diagnosis of osteoid osteoma was clearly confirmed by a subsequent CT scan (Figure 4).

**Figure 5:**
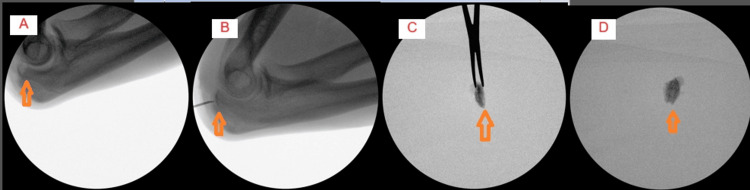
Intra-operative Images. (A-D) Osteoid osteoma location and excision under fluoroscopy (arrows).

Once the diagnosis was made, an open surgical excision was planned. Under general anesthesia and through a middle-line posterior approach, a limited split of the triceps was performed. The tip of the olecranon process was removed in the negative margin under C-arm guidance with the use of an osteotome (Figure 5). The olecranon was curated, and the joint was washed out.

Postoperatively, the patient had an uneventful recovery with immediate relief of symptoms. Early range of motion exercises were encouraged directly after surgery. Histologic examination confirmed the diagnosis of osteoid osteoma. The patient had a full return to his daily life activities six weeks postoperatively and returned to sports three months postoperatively.

## Discussion

Osteoid osteomas are frequently localized in femoral and tibial diaphysis or metaphysis [2,3]. The commonest intraarticular location of osteoid osteoma is in the hip. Diagnosis can be very challenging when it is located very close or within a joint [4,5], causing joint pain, stiffness, swelling, joint effusion and mimicking pathologies such as tendinitis, synovitis, chondral lesions and trauma [1,8,9,12]. Due to intraarticular prostaglandins, an intraarticular osteoma may have atypical clinical presentation without the classic nocturnal element of pain and with very minimal improvement with NSAIDs [1]. Elbow location is rare, and there are only a few reports of an osteoid osteoma found in the olecranon [13-15]. To the best of our knowledge, an osteoid osteoma has never been reported at the tip of the olecranon process in an intraarticular position.

It has been shown in the literature that diagnosis of intraarticular osteoid osteomas is usually made late at a mean of almost two years after the onset of symptoms [7,9]. Imaging studies, including standard X-rays, MRI, and CT scans, are of significant diagnostic value. Despite the fact that radiographic findings might be diagnostic in typically located diaphyseal lesions, classic X-rays are usually inconclusive in intraarticular tumors due to overlapping anatomical landmarks [7,16]. In the present case, extensive bone marrow oedema found in the MRI and athletic activities were two additional factors that further delayed the diagnosis, as the nidus was not visible in the first instance. Moreover, the fact that, in the present case, MRI without contrast was employed in all instances was a weakness of our diagnostic approach. French et al. reported that dynamic post-contrast MRI increases nidus conspicuity in cases with osteoid osteoma in atypical locations, allowing for even better accuracy in diagnosis than a CT scan [16]. 

As described in the literature, once the diagnosis was made, the excision of the lesion resulted in complete recovery with no pain around the elbow, full range of motion, and unrestricted return to everyday and athletic activity.

## Conclusions

An osteoid osteoma located close to a joint may be challenging to diagnose. In the case of an intraarticular location, the presentation may be confusing, mimicking trauma, tendinitis, and even chondral or osteochondral lesions. This can cause a delay in the diagnosis and prolong discomfort, pain, and disability. Excision or ablation of the lesion typically leads to a complete recovery.
